# Tianlongkechuanling Inhibits Pulmonary Fibrosis Through Down-Regulation of Arginase-Ornithine Pathway

**DOI:** 10.3389/fphar.2021.661129

**Published:** 2021-04-22

**Authors:** Lili Zhang, Sihao Qu, Lu Wang, Chunguo Wang, Qinghe Yu, Zhimin Zhang, Yirui Diao, Binbin Zhang, Yadong Li, Yuanyuan Shi, Peng Wang

**Affiliations:** ^1^ School of Life Sciences, Beijing University of Chinese Medicine, Beijing, China; ^2^ School of Chinese Medicine, Beijing University of Chinese Medicine, Beijing, China; ^3^ Traditional Chinese Medicine, First Affiliated Hospital of Guangzhou Medical University, Guangzhou, China; ^4^ Shenzhen Research Institute, Beijing University of Chinese Medicine, Shenzhen, China

**Keywords:** proteomics, tianlong kechuanling, pulmonary fibrosis, arginase, arginase-ornithine pathway

## Abstract

**Background:** Pulmonary Fibrosis (PF) is an interstitial lung disease characterized by excessive accumulation of extracellular matrix in the lungs, which disrupts the structure and gas exchange of the alveoli. There are only two approved therapies for PF, nintedanib (Nib) and pirfenidone. Therefore, the use of Chinese medicine for PF is attracting attention. Tianlongkechuanling (TL) is an effective Chinese formula that has been applied clinically to alleviate PF, which can enhance lung function and quality of life.

**Purpose:** The potential effects and specific mechanisms of TL have not been fully explored, yet. In the present study, proteomics was performed to explore the therapeutic protein targets of TL on Bleomycin (BLM)-induced Pulmonary Fibrosis.

**Method:** BLM-induced PF mice models were established. Hematoxylineosin staining and Masson staining were used to analyze histopathological changes and collagen deposition. To screen the differential proteins expression between the Control, BLM, BLM + TL and BLM + Nib (BLM + nintedanib) groups, quantitative proteomics was performed using tandem mass tag (TMT) labeling with nanoLC-MS/MS [nano liquid chromatographymass spectrometry]). Changes in the profiles of the expressed proteins were analyzed using the bioinformatics tools Gene Ontology (GO) and the Kyoto Encyclopedia of Genes and Genomes (KEGG). The protein–protein interactions (PPI) were established by STRING. Expressions of α-smooth muscle actin (α-SMA), *Collagen I* (*Col1a1*), *Fibronectin* (*Fn1*) and enzymes in arginase-ornithine pathway were detected by Western blot or RT-PCR.

**Result:** TL treatments significantly ameliorated BLM-induced collagen deposition in lung tissues. Moreover, TL can inhibit the protein expressions of α-SMA and the mRNA expressions of *Col1a1* and *Fn1*. Using TMT technology, we observed 253 differentially expressed proteins related to PPI networks and involved different KEGG pathways. Arginase-ornithine pathway is highly significant. The expression of *arginase1 (*Arg*1), carbamoyltransferase (OTC), carbamoy-phosphate synthase (CPS1), argininosuccinate synthase (ASS1), ornithine aminotransferase (OAT) argininosuccinate lyase (ASL) and inducible nitric oxide synthase (iNOS)* was significantly decreased after TL treatments.

**Conclusion:** Administration of TL in BLM-induced mice resulted in decreasing pulmonary fibrosis. Our findings propose that the down regulation of arginase-ornithine pathway expression with the reduction of arginase biosynthesis is a central mechanism and potential treatment for pulmonary fibrosis with the prevention of TL.

## Introduction

Pulmonary Fibrosis (PF) is a chronic, progressive fibrotic interstitial lung disease, characterized by the excessive accumulation of extracellular matrix and fibrotic tissue in the lungs (Lederer and Martinez, 2018; Sgalla et al., 2018). It leads to decreased lung compliance, disrupted gas exchange, and ultimately respiratory failure and death (Raghu et al., 2011; Richeldi et al., 2017). There are only two approved therapies for PF, nintedanib (Nib) and pirfenidone, which can reduce the rate of progression of fibrosis (Taniguchi et al., 2010; Wongkarnjana et al., 2019). But they also have some uncertainties, side effects and poor prognosis. Therefore, it may be a good choice to seek treatments from Chinese medicine.

Tianlongkechuanling (TL) is a traditional Chinese medicine compound preparation, which composed of *Houttuynia cordata Thunb (*Saururaceae *F. Voigt), Tussilago farfara L (*Asteraceae *Bercht. and J. Presl), Pinellia ternata (Thunb.) Makin (*Araceae *Juss.), Sinapis alba L (*Brassicaceae *Burnett), Scutellaria baicalensis Georgi (*Lamiaceae *Martinov), Ephedra sinica Stapf (*Ephedraceae *Dumort.), Cyperus rotundus L (*Aristolochiaceae *Juss.), Schisandra chinensis (Turcz.) Baill (*Schisandraceae *Blume), Semiaquilegia adoxoides (DC.) Makino (*Ranunculaceae *Juss.), and Asarum sieboldii Miq (*Aristolochiaceae *Juss.).* It has been used to treat chronic cough, chronic bronchitis, chronic obstructive pulmonary disease, asthma, and other chronic respiratory diseases in clinical, and achieved good therapeutic effects (Mai and Xia, 2009; Zhu et al., 2007; Qiu et al., 2000; Zhang et al., 2008). Previous studies also confirmed that TL can reduce the expression of α -SMA induced by transforming growth factor (TGF-β1) in fibroblasts (Zhang et al., 2007). And some ingredients of the TL Extract were identified that can alleviate TGF β1-stimulated the production of type I collagen to inhibit collagen deposition in fibroblast cells (Wang et al., 2018; Wang et al., 2016). As we know TGF-β1 can enhance the expression of the lung matrix that causes PF (Ayed et al., 2017; Saito et al., 2018; Chanda et al., 2019). It has been reported that TGF-β1 is a crucial cytokine of arginase-inducing (Endo et al., 2003; Torsello et al., 2016). And a previous study confirmed that TGF-β1 could increase arginase activity and expression in both fibroblasts and lung tissue (Liu et al., 2005). Increased arginase expression contributed to the development of pulmonary fibrosis (Endo et al., 2003; Kitowska et al., 2008; Maarsingh et al., 2008). The inhibition of arginine metabolism has been considered a new therapeutic approach to reduce and slow the progression of PF (Gao et al., 2019). Therefore, we analyze whether TL can inhibit pulmonary fibrosis through down-regulating arginine metabolism by using quantitative proteomics to study the mechanism of TL treatment of pulmonary fibrosis.

Large-scale and systematic protein analysis can help us understand the physiology and pathology of animals, but this process is undoubtedly extremely complicated. In recent years, due to the development of mass spectrometry technology, proteomics technology has made considerable progress (Cheung and Juan et al., 2017; Palmfeldt and Bross et al., 2017). Quantitative proteomics provides us with different protein abundances under different conditions of animals, and further provides the information of molecular interactions and signaling pathways, which can help us find the occurrence, development and treatment mechanisms of diseases (Cifani and Kentsis et al., 2017; Zhang et al., 2019). In the present study, we evaluated the potential protective effect, and explored the underlying mechanism of TL in BLM-induced PF through proteomics analysis, which involved arginase-ornithine pathway.

## Materials and Methods

### Preparation of Tianlongkechuanling

Constituent drugs of TL were purchased from Daxiang Group (Guangdong, China):

*Houttuynia cordata Thunb (*Saururaceae *F. Voigt), Tussilago farfara L (*Asteraceae *Bercht. and J. Presl), Pinellia ternata (Thunb.) Makin (*Araceae *Juss.), Sinapis alba L (*Brassicaceae *Burnett), Scutellaria baicalensis Georgi (*Lamiaceae *Martinov), Ephedra sinica Stapf (*Ephedraceae *Dumort.), Cyperus rotundus L (*Aristolochiaceae *Juss.), Schisandra chinensis (Turcz.) Baill (*Schisandraceae *Blume), Semiaquilegia adoxoides (DC.) Makino (*Ranunculaceae *Juss.) and Asarum sieboldii Miq (*Aristolochiaceae *Juss.).* After soaking the TL in water and decocting for 30 min, we filtered the medicine residue and got the medicine liquid. Then a rotary evaporator was used to concentrate to obtain an extract. We put it in the refrigerator at −20°C for 24 h. After treatment with vacuum freeze dryer, it was sterilized and stored in a refrigerator at 4°C. We diluted it with distilled water when needed.

### Chromatographic and Mass Spectrometric Conditions

We have performed HPLC analyses for the identification of chemical fingerprints presented in TL freeze dryer. The UPLC system was coupled with a LTQ-Orbitrap XL (Thermo Fisher Scientific, SanJose, CA, United States) equipped with an electrospray ionization source (ESI) and controlled by Xcalibur software (Version 2.1). Dionex Utimate 3000 UHPLC Plus Focused Ultra High Liquid Chromatography System included binary pumps, a autosampler, a Column thermostat and DAD detector (Thermo Fisher Scientific, SanJose, CA, United States). 0.45 μm microporous membrane (Tianjin Jinteng Experimental Equipment Co., Ltd., Tianjin, China). Sartorious BT 25S electronic analytical balance (Beijing Sartorius Instrument Co., Ltd., Beijing, China). Ultrasonic cleaner (Beijing Zhongshengming Technology Co., Ltd., Beijing, China).

In positive ion mode, nitrogen was used as the sheath gas and auxiliary gas, and flow rates were set at 40 arbitrary units and 20 arbitrary units respectively. The capillary voltages were set to 35.0 V, the source temperature was set to 350°C, the source voltages was set to 4 kV, and the tube lens was set to 110 V. In negative ion mode, nitrogen was used as the sheath gas and auxiliary gas, and flow rates were set at 30 arbitrary units and 10 arbitrary units respectively. The capillary voltage was set to 35.0 V, the source temperature was set to 350°C, the source voltage was set to 3 kV, and the tube lens was set to 110 V in negative ion mode.

The column adopted was octadecylsilane chemically bonded silica (Kromasil 100–5, C18 250 × 4.6 mm 5 μm). Mobile phase A was acetonitrile mobile phase B is an aqueous solution of 0.05% phosphoric acid. The flow rate adopted was 0.8 ml/min and the column temperature was set at 30°C. Elution conditions are summarized in Table 1.

**TABLE 1:** Elution conditions.

Time (min)	% Mobile phase A (acetonitrile)	% Mobile phase A (0.0.5% phosphoric acid in water)
0∼8	19	81
8∼35	19→50	81→50
35∼40	50→80	50→20
40∼50	80→50	20→50
55∼60	19	81

### Bleomycin-Induced Lung Fibrosis and Drug Treatment

Forty-eight, 8 week-old wild-type C57BL/6 male mice were purchased from Beijing SPF Biotechnology Co., Ltd (Beijing, China). Animal certificate number: 11401500028627. All of the animal procedures including housing, care and experimental protocols were approved by the Animal Care and Use Committee of Beijing University of Traditional Chinese Medicine (BUCM-4-2019102105-4119). All mice were randomly divided into five groups after one-week habituation. Control group, Tianlongkechuanling control group (TL group), model group (BLM group), Tianlongkechuanling treatment group (BLM + TL group), nintedanib treatment group (BLM + Nib group). BLM was purchased from Nippon Kayaku (Batch number 970592). After the mice were anesthetized. The mice of the Control group and the TL group were intratracheally instilled with 100 µL 0.9% saline and the remaining groups were intratracheally instilled with 100 ul bleomycin solutions (2.5 mg/kg in 0.9% NaCl) to prepare a pulmonary fibrosis model. After the model was done, mouse weight was recorded. The day of modeling was day 0. After 14 days of BLM treatment, TL (45 mg/kg), and Nib (60 mg/kg, positive drug) were administrated intragastrically once a day for 14 days. Mice in the Control group and the model group were intragastrically conducted with 0.9% saline. After 28 days, all mice were killed, and lung tissues were weighted. Left lung tissue was fixed for pathology and in 4% paraformaldehyde over 24 h, and the rest was frozen for subsequent testing.

### Histopathological Examination

For histological analysis, pulmonary tissues were fixed with 4% paraformaldehyde and embedded in paraffin. The paraffin blocks were cut at 5 μm using microtome. Sections stained against hematoxylin and eosin (H&E) or Masson Trichrome (Sinopharm Chemical Reagent Beijing Co., Ltd., Beijing, China). Inflammation and collagen deposition of the sections were observed and assessed with an optical microscope.

### Calculation of Lung Coefficient and Determination of Hydroxyproline Content

Lung coefficient was the ratio of lung tissue weight to body weight. The right lower lobe of the mice was homogenized to determine hydroxyproline content using Hydroxyproline detection kit (Jian Cheng Biological Engineering Institute, Nanjing, China) according to the manufacturer’s instructions.

### Western Immunoblot Analysis

Total protein was extracted from frozen lung tissue using RIPA Lysis Buffer (Applygen, Beijing, China) supplemented with protease/phosphatase inhibitor cocktail. The protein concentrations were determined with a BCA protein quantification kit (Applygen, Beijing, China). The lysates were mixed with 8% SDS-PAGE and then electrophoresis was performed. The proteins were then transferred onto polyvinylidene fluoride membranes (Bio-Rad) and were blocked with 5% skim milk for 2 h at room temperature. The membranes were incubated with primary antibodies against a-SMA (1:1000), Arg1 (1:1000), OTC (1:1000), CPS1 (1:2000), ASS1 (1:1000), and GAPDH (1:40,000, all from Abcam, Cambridge, MA, United States) overnight at 4°C. Subsequently, the blots were incubated for 1 h at room temperature with secondary antibodies (1:5000; Abcam). The protein bands were detected using electrochemiluminescence (ECL) reagent (Tanon, Shanghai, China) and the intensities of the signals were quantitatively analyzed using ImageJ Software.

### Quantitative Real-Time PCR (qRT-PCR)

Lung tissues were used for real-time RT-qPCR. Total RNA was extracted using a RNeasy^®^ Lipid Tissue Mini Kit (QIAGEN, Valencia, CA, United States). RNA quality and quantity were determined using a spectrophotometer (Thermo Nano Drop™ 2000c, United States) and denaturing agarose gel electrophoresis. *Col1a1*, *Fn1*, *OAT, ASL, iNOS,* and *GAPDH* genes were amplified using the forward and reverse primers listed in Table 2. Complementary DNA was synthesized from RNA (2 μg), using Reverse Transcription Master Mix (QIAGEN, Valencia, CA, United States). RT-qPCR was performed using SYBR Green PCR Master Mix (QIAGEN, Valencia, CA, United States) on a CFX-96 system. All the sample data were quantified using the comparative 2 ^ -delta Ct method and presented as the mean ratio to *GAPDH*. The experiment was performed in triplicate.

**TABLE 2:** Primers used for real-time polymerase chain reaction.

Gene name	Forward primer	Reverse primer
*Col1a1*	CGA​CCT​CAA​GAT​GTG​CCA​CT	GCA​GTA​GAC​CTT​GAT​GGC​GT
*Fn1*	CCC​CAA​CTG​GTT​ACC​CTT​CC	GGT​TGG​TGA​TGA​AGG​GGG​TC
*OAT*	CCC​TCT​GAC​GTT​GTG​ACC​TC	GCA​CAC​CTT​CCA​AGC​ATC​AC
*ASL*	AAG​TGG​AGC​CCT​GAA​GAA​ACC	AAA​TCC​CCC​AGC​CCA​CTC​AT
*iNOS*	CTG​GGA​GCG​CTC​TAG​TGA​AG	GGG​GAG​CCA​TTT​TGG​TGA​CT
*GAPDH*	CTC​TGG​TGG​CTA​GCT​CAG​AAA	CCC​TGT​TGC​TGT​AGC​CGT​AT

## Tandem Mass Tag Proteomics Analysis

### Protein Extraction and Tandem Mass Tag Labeling

Three lung tissues were randomly selected from Control group, BLM group, BLM + TL group, and BLM + Nib group for proteomics experiments. For protein extraction, lung tissues from mice were respectively homogenized and lyzed in lysis buffer (Beijing Bangfei Biological Technology Co., Ltd.). Then, the homogenate was centrifuged at 13,300 r/min for 10 min at 4°C. The supernatant, which contained proteins, was recovered. The protein concentration was measured by BCA (bicinchoninic acid) kit (Thermo Fisher Scientific, SanJose, CA, United States) according to the manufacturer’s protocol.

100 μg peptide mixture of each sample was labeled by TMT reagent according to the manufacturer’s instructions (Thermo Fisher Scientific, SanJose, CA, United States).

### Peptide Fractionation With Reversed Phase Chromatography

TMT labeled peptides were fractionated by RP chromatography using HPLC. The peptide mixture was diluted with buffer A (2% ACN, pH 10.0) and loaded onto xBridge peptide BEH 130 C18 column (Waters). The peptides were eluted at a flow rate of 0.7 ml/min with buffer B (98% ACN, pH 10.0). The collected fractions were dried down via vacuum centrifugation at 45°C.

### Mass Spectrometry Analysis

#### 1) Easy nLC

Each fraction was injected for nanoLC-MS/MS analysis. The peptide mixture was loaded onto the C18-reversed phase analytical column (Thermo Fisher Scientific, SanJose, CA, United States) in buffer A (0.1% formic acid) and separated with a linear gradient of buffer B (80% acetonitrile and 0.1% formic acid) at a flow rate of 300 nL/min.

#### 2) LC-MS/MS Analysis

LC-MS/MS analysis was performed on a Q Exactive Plus mass spectrometer (Thermo Fisher Scientific, SanJose, CA, United States) that was coupled with Easy nLC (Thermo Fisher Scientific) for 90 min. The mass spectrometer was operated in positive ion mode. MS data was acquired using a data-dependent top10 method dynamically choosing the most abundant precursor ions from the survey scan (350–1800 m/z) for HCD fragmentation. Automatic gain control (AGC) target was set to 3e6, and maximum inject time to 45 ms. Survey scans were acquired at a resolution of 70,000 at m/z 200 and resolution for HCD spectra was set to 17,500 at m/z 200, and isolation width was 2 m/z. Normalized collision energy was 30 eV.

### Sequence Database Search and Data Analysis

MS/MS raw files were processed using Proteome Discoverer 2.1 (ver.2.1 Thermo Fisher Scientific, SanJose, CA, United States) software was used for data analysis, and searched against the human proteome database, downloaded on Uniprot. Decoys for the database search were generated with the revert function. The following options were used to identify the proteins: Peptide mass tolerance = ± 15 ppm, MS/MS tolerance = 0.02 Da, enzyme = trypsin, missed cleavage = 2, fixed modification: TMT6pLex (N-term), TMT6pLex (K), Carbamidomethyl (C), variable modification: Oxidation (M), Acetyl (Protein N-term). The false discovery rate (FDR) for peptides and proteins was set to 0.01. The upregulated or downregulated proteins in both replicates with relative quantification *p*-values < 0.05 and 1.5-fold changes were selected as being differentially expressed in the data. The proteomics data have been deposited to the ProteomeXchange Consortium via the PRIDE partner repository with identifier PXD024058.

## Results

### HPLC Analysis Identified Ingredients in the TL Extract

The mass spectra were recorded across the range of m/z 120–1800, with accurate mass measurements of all of the mass peaks. Accurate mass measurements of each peak from the total ion chromatogram (TIC) were obtained by means of ESI source. All of the data were processed using Xcalibur software version 2.7.

**FIGURE 1:**
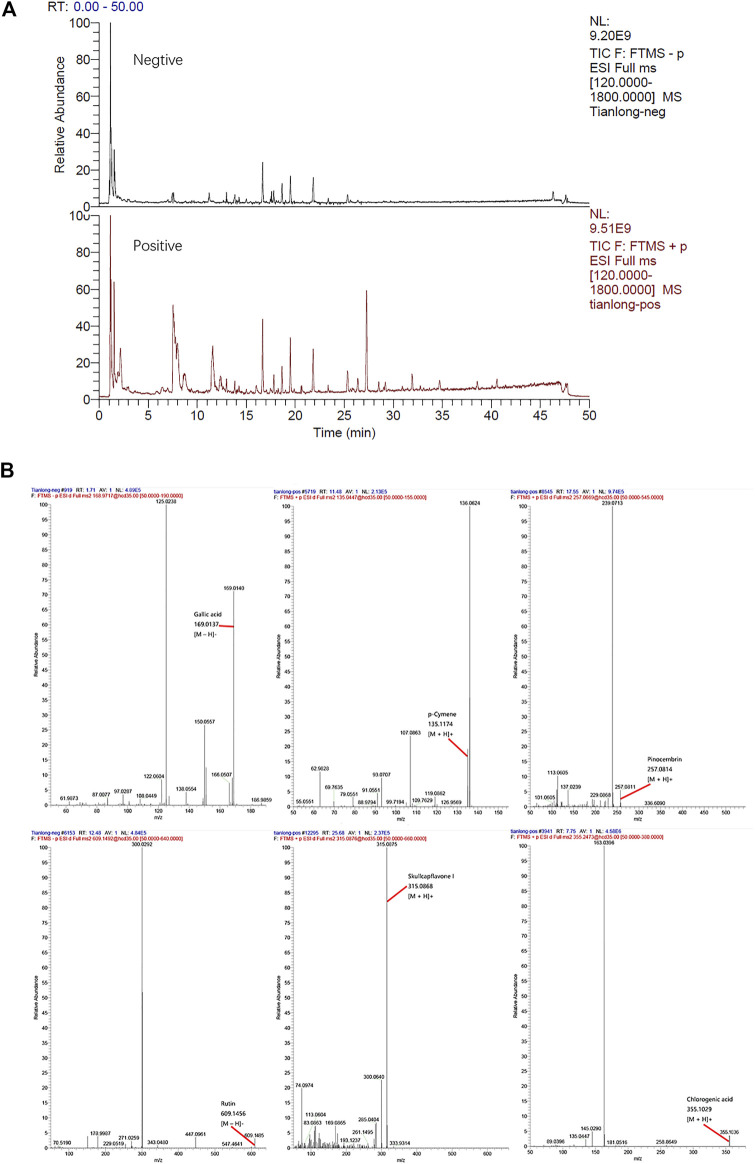
**(A)** Total ion chromatogrm monitored in positive and negative ion modes for TL extract **(B)** TL extract composition analysis: TL has Gallic acid, *p*-Cymene, Pinocembrin, Rutin, Skullcapflavone I, Chlorogenic acid, and other ingredients.

Comparing the information collected from the literature and standards with the data obtained by the HPLC-LTQ-Orbitrap MS method mass spectrometry, a total of 57 compounds were identified from the TL extract, including Gallic acid, *p*-Cymene, Pinocembrin, Rutin, Skullcapflavone Ⅰ, Chlorogenic acid, and other ingredients (Figure 1, Supplementary Table S1).

### TL Alleviated Bleomycin-Induced Pulmonary

**FIGURE 2:**
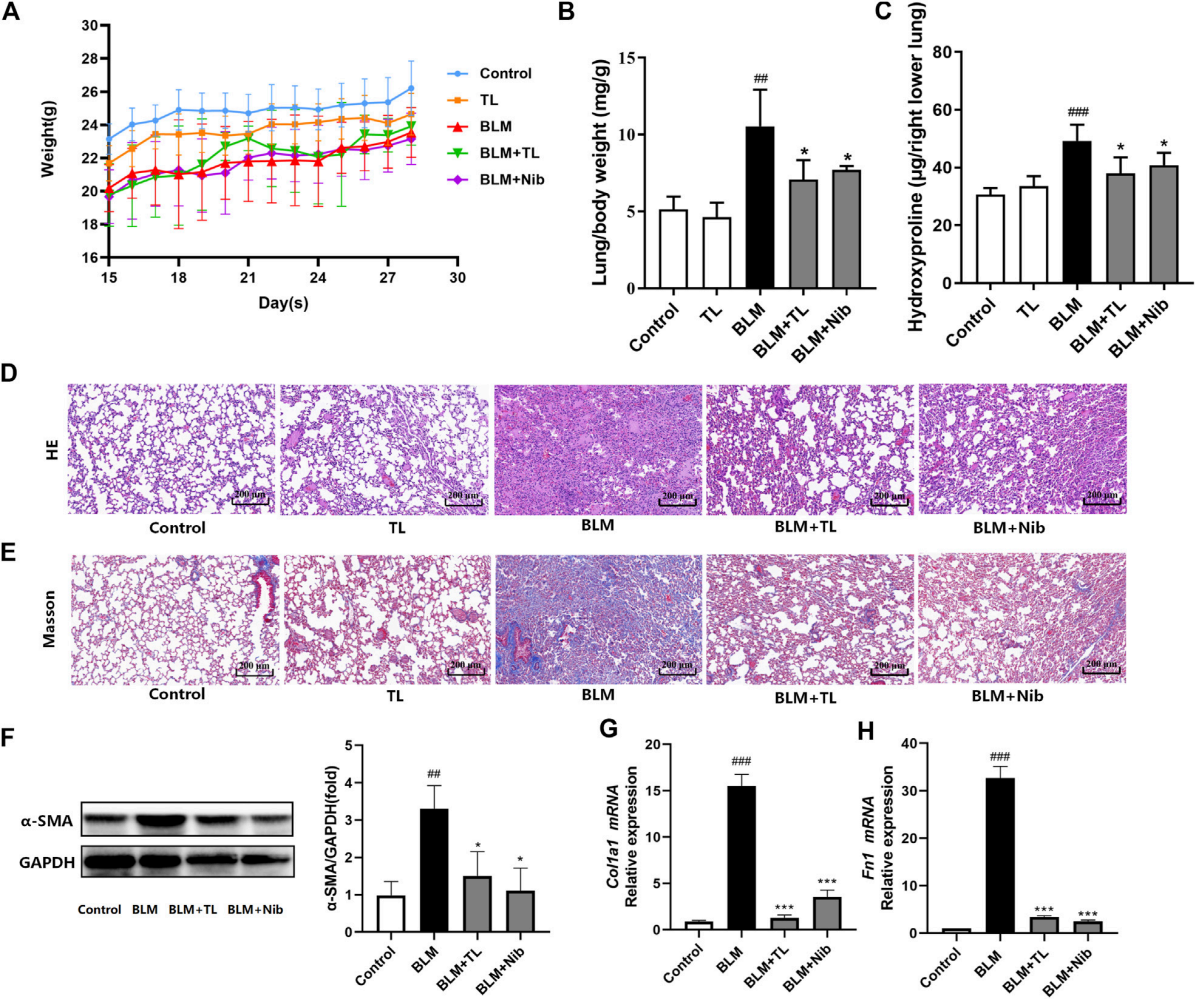
TL-treated mice are protected against BLM-induced pulmonary fibrosis. After 14 days of BLM (2.5 mg/kg) or 0.9% saline treatment, mice were orally administrated with TL (45 mg/kg), Nib (nintedanib, 60 mg/kg, positive drug) or 0.9% saline once a day for 14 days **(A)** changes of body weight of mice (*n* = 5–10) **(B)** lung coefficient (*n* = 5) **(C)** quantification of hydroxyproline contents (*n* = 5) **(D-E)** H and E and Masson’s trichrome staining section of lung tissue (*n* = 3) **(F)** the expression of *α-SMA* in lung tissue were detected by western blot and densitometrically quantified data of *α-SMA/GAPDH* expression ratio (*n* = 3) **(G-H)** relative mRNA expression of *Col1a1* and *Fn1* were detected using real time-PCR analysis (*n* = 3). Data were expressed as mean ± SEM, vs. the Control group, ^*#*^ *p* < 0.05, ^*##*^ *p* < 0.01, ^*####*^ *p* < 0.001, vs. the BLM group *p < 0.05, **p < 0.01, ***p < 0.001.

The anti-pulmonary fibrosis effects of TL on the BLM-induced pulmonary fibrosis are shown in Figure 1. Comparing with the control group, a significant loss of body weight? and increase in the lung index and hydroxyproline content were observed in BLM group mice (*p* < 0.01). But, BLM-induced weight loss and the increase in the lung index (*p* < 0.001) and hydroxyproline content (*p* < 0.01) were reversed by TL treatments (Figures 2A–C). H&E staining and Masson’s trichrome staining showed pronounced diffuse fibrosis, inflammatory cells infiltration, edema, structure destruction and a great amount of collagen deposition in BLM group as compared with the control group. Whereas, fibrosis showed apparent symptomatic relief in BLM + TL group and BLM + Nib group compared to the BLM group (Figures 2D,E). α-Smooth muscle actin is a marker of myofibroblasts that plays an important role in the development of the fibrotic lesion (Ding et al., 2019). Comparing with controls, the expression of α-Smooth muscle actin (α-SMA) detected by western blotting in the BLM group was increased, while the levels of α-SMA significantly decrease in the BLM + TL group and the BLM + Nib group (Figure 2F). The main feature of PF is the deposition of extracellular matrix (ECM), while *Collagen I* (*Col1a1*) is the main source of ECM and is secreted in large quantities by myofibroblasts (Zhang et al., 2019). *Fibronectin 1* (*Fn1*) can mediate the adhesion between cells and the adhesion between cells and matrix (Halper and Kjaer, 2014). Unsurprisingly, qRT-PCR of *Col1a1,* and *Fn1* also indicated that the TL treated mice exhibited a lower degree of lung fibrosis than the BIM treated mice (Figures 2G,H). These results suggest that TL effectively attenuated BLM-induced pulmonary fibrosis.

### Quantitative Proteomics Analyses to Reveal Pulmonary Fibrosis Proteins

We next sought out to determine the molecular mechanisms in which TL inhibits fibrosis using TMT Quantitative Proteomics.

### Heatmap Analysis

**FIGURE 3:**
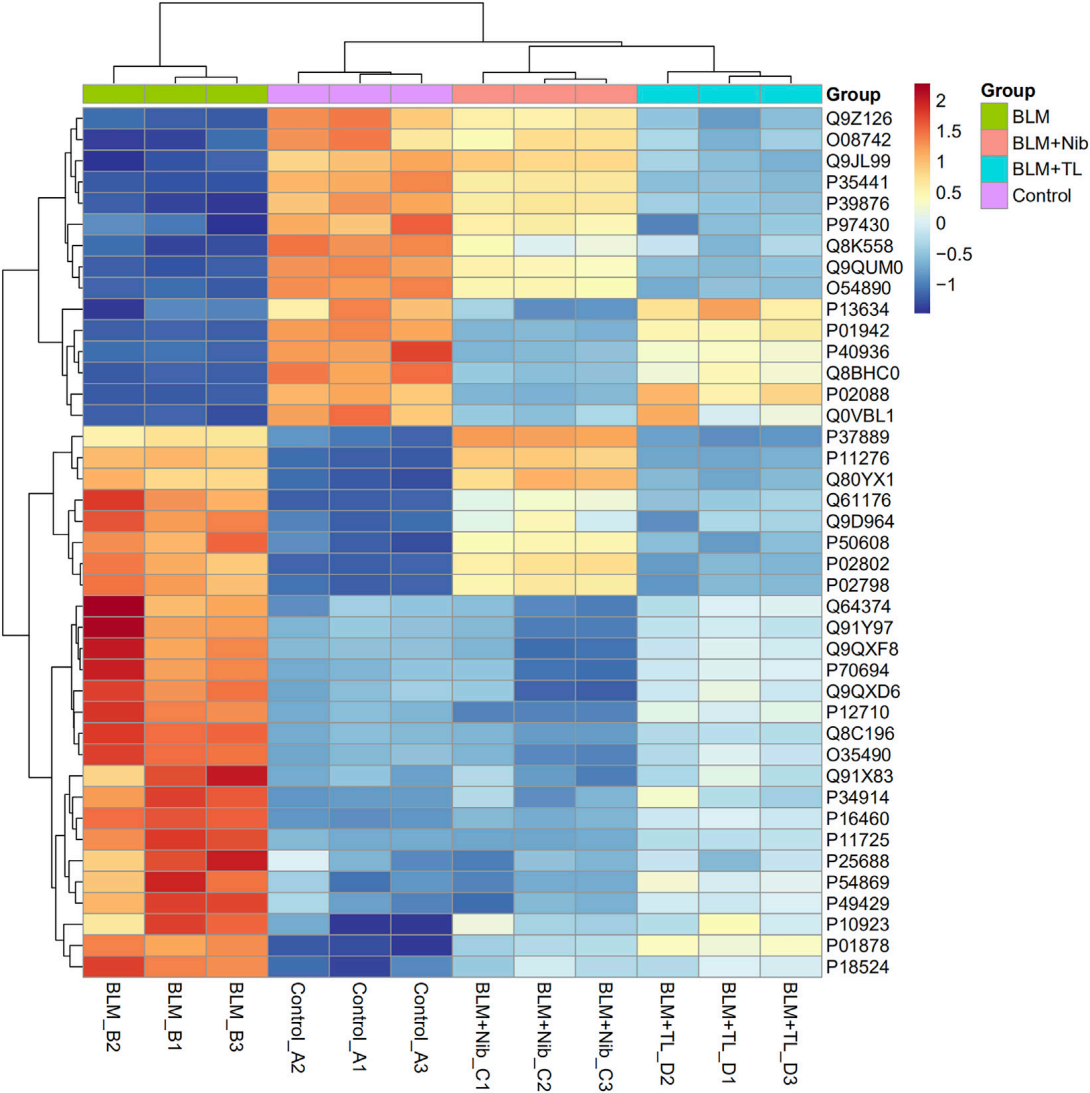
Heatmap of the levels of differentially expressed proteins. The red-colored clusters represent up-regulated proteins, and the blue-colored clusters represent down-regulated proteins. The intensity of the color represents the degree of up-regulation and down-regulation. The expression of Arg1 (Q61176), OTC (P11725), and ASS1 (P16460) in BLM group were reversed after TL treatment.

Hierarchical clustering was performed for all proteins as well as differentially expressed proteins to test if the groups (BLM group, Control group, BLM + Nib group and BLM + TL group proteins (*p* < 0.05, vs. the Mod group)) can be separated by unsupervized statistical techniques. The three horizontal clusters represent the technical replicates (Figure 3). When significantly differing proteins were used for clustering the sample groups, there was good separation between BLM group and Control group; however, few samples overlapped into each other’s group. Mostly similar protein levels were observed in Control group, BLM + Nib group, and BLM + TL group compared with BLM group. Most of differing proteins were related to ECM-receptor interaction and metabolic pathways. Our data demonstrated that the expression of Arg1 (Q61176), OTC (P11725), ASS1 (P16460), and other enzymes related to arginine metabolism in BLM group was reversed after TL or nintedanib treatment.

### Gene Ontology Analysis

**FIGURE 4:**
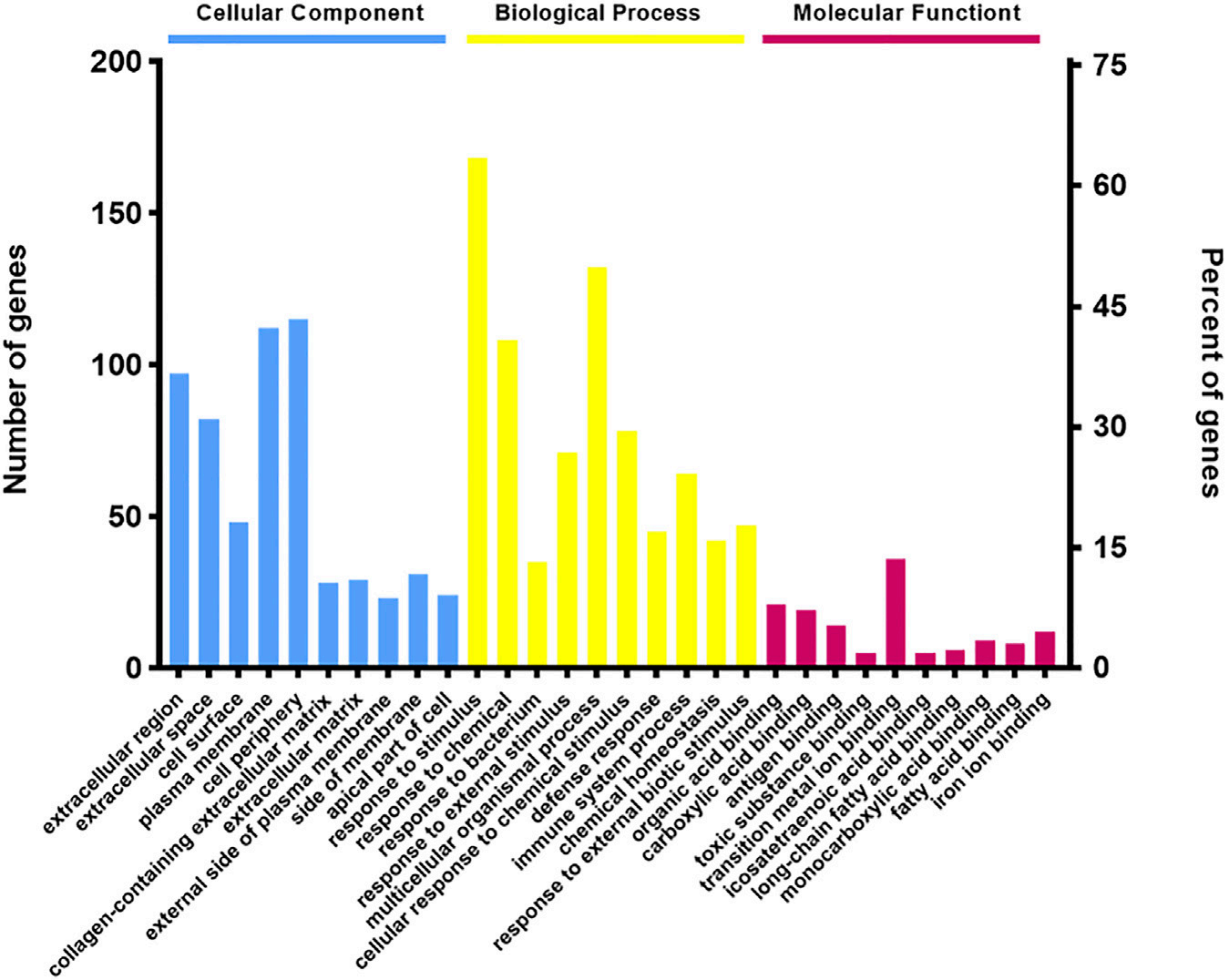
Gene Ontology (GO) term performance analysis of differentially regulated proteins between the BLM + TL group and BLM group. An overview of the GO annotations of top 10 significantly enriched terms in three categories: cellular compartments, biological processes, and molecular functions (*p* < 0.05). Terms in the same category were ordered based on the *p*-values, increased from left to right. Information for the numbers of involved genes/proteins and percentages in a term is provided on the left and right vertical axes.

Gene Ontology (GO) analysis showed the overviews of dysregulated proteins in the biological process (BP), cell component (CC), and molecular function (MF) categories. An overview of the GO annotations of top 10 significantly enriched terms in three categories. The CC analysis showed that most of the identified proteins belonged to extracellular region, collagen-containing extracellular matrix, extracellular matrix and external side of plasma membrane. In the BP analysis, the majority of identified proteins classified into response to stimulus, multicellular organismal process, response to chemical, and response to external stimulus. The MF classification revealed that most of these proteins were involved in transition metal ion binding, organic acid binding, carboxylic acid binding, and antigen binding (Figure 4). GO analysis indicated that differentially expressed proteins exhibited cellular distributions in extracellular region and collagen-containing extracellular matrix, consistent with the fact that pulmonary fibrosis associated with collagen deposition in lung extracellular matrix (ECM). Furthermore, our data demonstrated that synthesis and metabolism of a wide variety of substances play a role in molecular function and biological process.

### Protein–Protein Interactions Network

**FIGURE 5:**
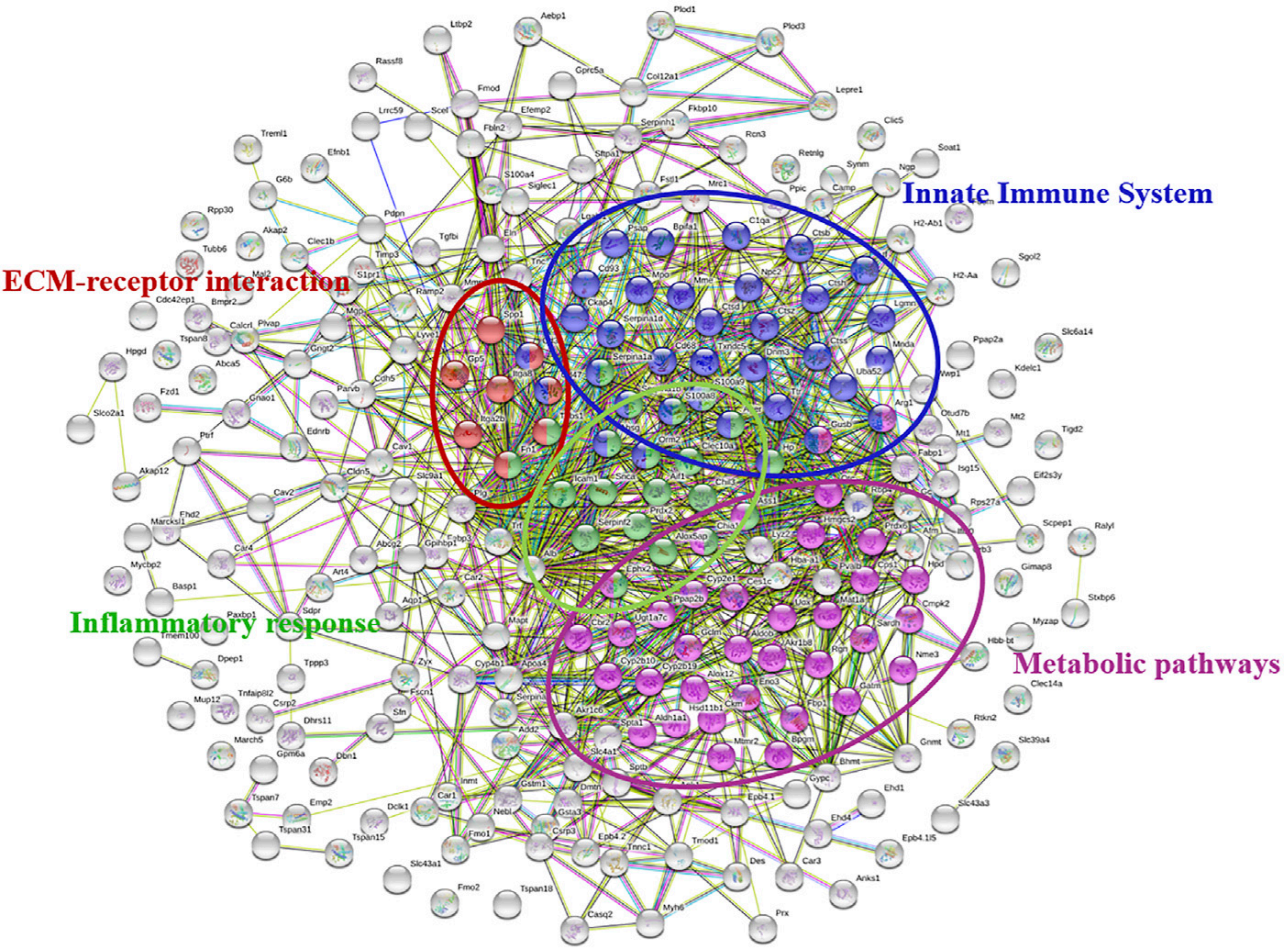
PPI network of differentially regulated proteins between the BLM + TL group and BLM group. Different colors represent different functions of genes. Genes with similar functions were grouped together. Metabolic pathways have the largest number of genes.

Comparing the proteomics results of the BLM-TL group and the BLM group, 253 different proteins were found. The String website was used to analyze the function of differential proteins and construct a PPI network. Most of the related differential proteins were mainly concentrated in four sub-network clusters: Metabolic pathways, ECM-receptor interaction, Innate Immune System and Inflammatory response. Among them, Metabolic pathways have the largest number of genes, showing high priority (Figure 5).

### Kyoto Encyclopedia of Genes and Genomes Pathway Analysis

**FIGURE 6:**
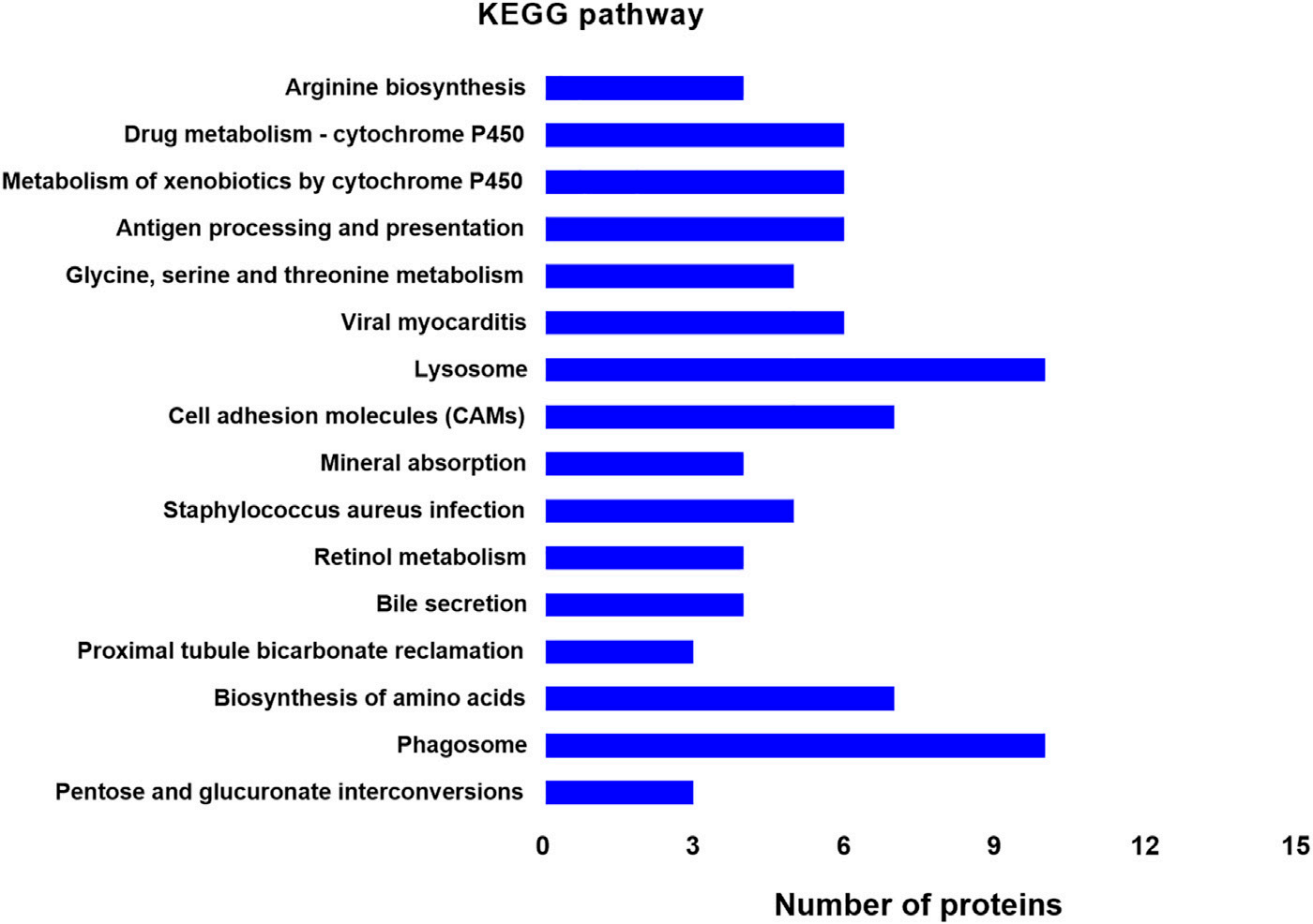
Distribution of enriched KEGG pathways between the BLM + TL group and BLM group. The vertical axis indicates the KEGG pathway. The horizontal axis presents the number of proteins. The *p*-values of enrichment analysis gradually decreases from top to bottom. Arginine biosynthesis shows the highest significance.

KEGG analysis revealed significant pathways with *p* < 0.05 (Figure 4, Mod + TL group vs. Mod group). The top five significantly enriched pathways were Arginine biosynthesis (ko00220), Drug metabolism-cytochrome P450 (ko00982), Metabolism of xenobiotics by cytochrome P450 (ko00980), Antigen processing and presentation (ko04612), Glycine, serine and threonine metabolism (ko00260) (Figure 6). These data suggest that the KEGG pathways were mainly associated with metabolism and Arginine biosynthesis was the most significant pathway, indicating Arginine biosynthesis and metabolism signaling pathway could be a novel target for the treatment of PF. These data are consistent with the PPI Network analysis.

### TL Inhibits the Arginine Biosynthesis and Metabolism Signaling Pathway

**FIGURE 7:**
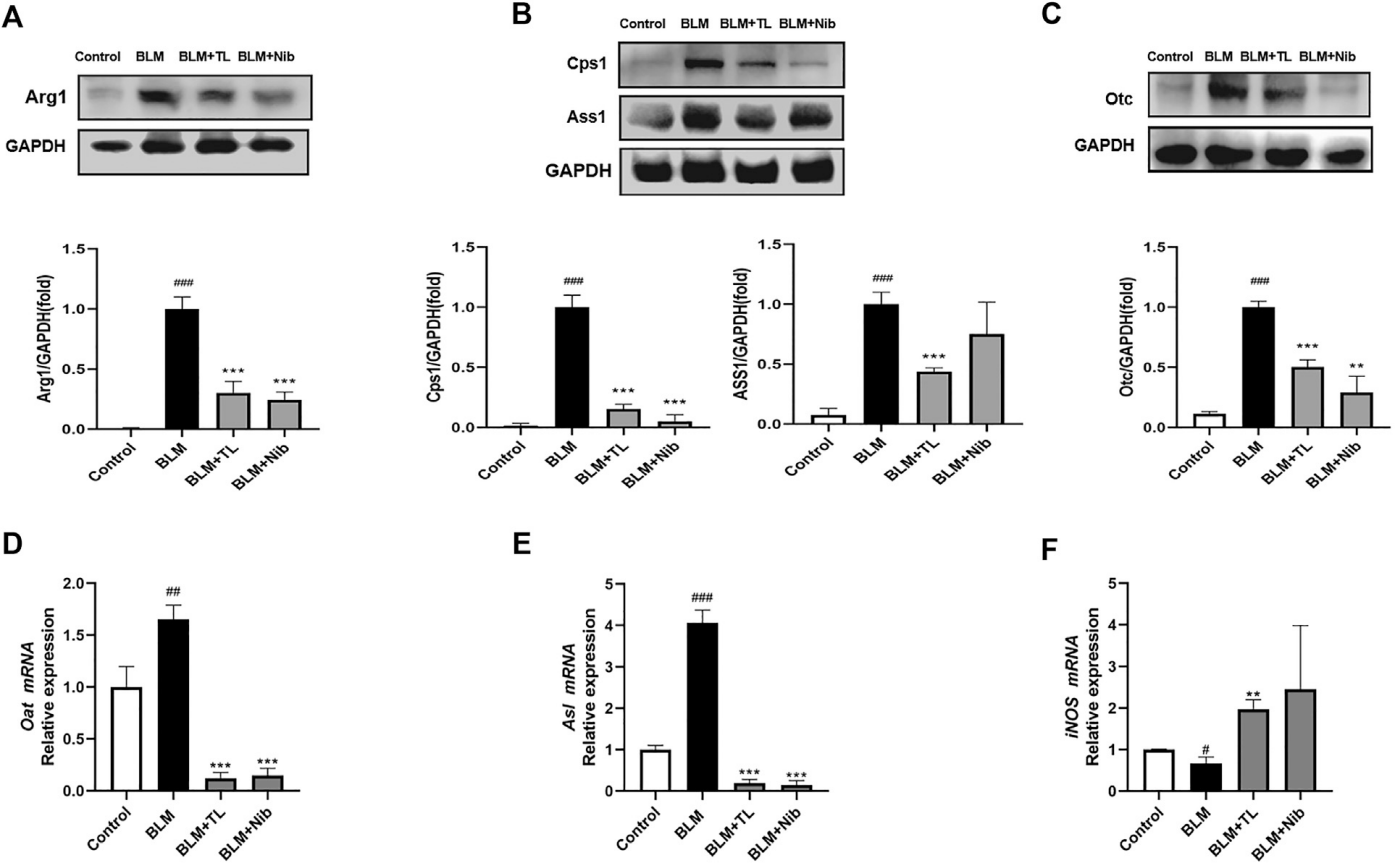
TL inhibited BLM-induced arginase-ornithine pathway upregulation *in vivo*. 14 days after BLM (2.5 mg/kg) or 0.9% saline treatment, Control and BLM mice were treated with 0.9% saline; TL mice were treated with 45 mg/kg/day TL; Nib mice were treated with 60 mg/kg/day Nib. Representative Western blotting images and fold changes in relative densitometric values of Arg*1, CPS1, ASS1, and OTC* in lung tissues from mice (*n* = 3) **(A, B, C).** Relative mRNA expression of *OAT, ASL, and iNOS* in lung tissues were detected by real time-PCR analysis (*n* = 3) (**D, E, F**). Data were expressed as mean ± SEM, vs. the Control group, ^*#*^ *p* < 0.05, ^*##*^ *p* < 0.01, ^*####*^ *p* < 0.001, NS, non-significant; vs. the BLM group *p < 0.05, **p < 0.01, ***p < 0.001, NS, non-significant.

Western blot was used to detect the expression of arginine-ornithine cycle-related proteins in the lung tissues of mice with BLM-induced pulmonary fibrosis. Arginase 1 (Arg1) is a key enzyme in the cycle and can metabolize arginine as ornithine. WB results showed that the content of Arg1 in the lung tissue of mice induced by BLM was significantly increased, and TL can significantly inhibit the expression of arginase 1(Arg1, Figure 7A), argininosuccinate synthase (ASS1, Figure 7B), carbamoyl phosphate synthase (CPS1, Figure 7B) and ornithine carbamoyl transferase (OTC, Figure 7C). In addition, qRT-PCR results showed that TL can greatly reduce the expression of ornithine aminotransferase (OAT, Figure 7D), argininosuccinate lyase (ASL, Figure 7E), and inducible nitric oxide synthase (iNOS, Figure 7F) at the mRNA level. These results proved that TL can significantly down-regulate the expression of arginase-ornithine cycle-related enzymes in BLM-induced mice lung tissues, thereby treating lung fibrosis.

## Discussion

**FIGURE 8:**
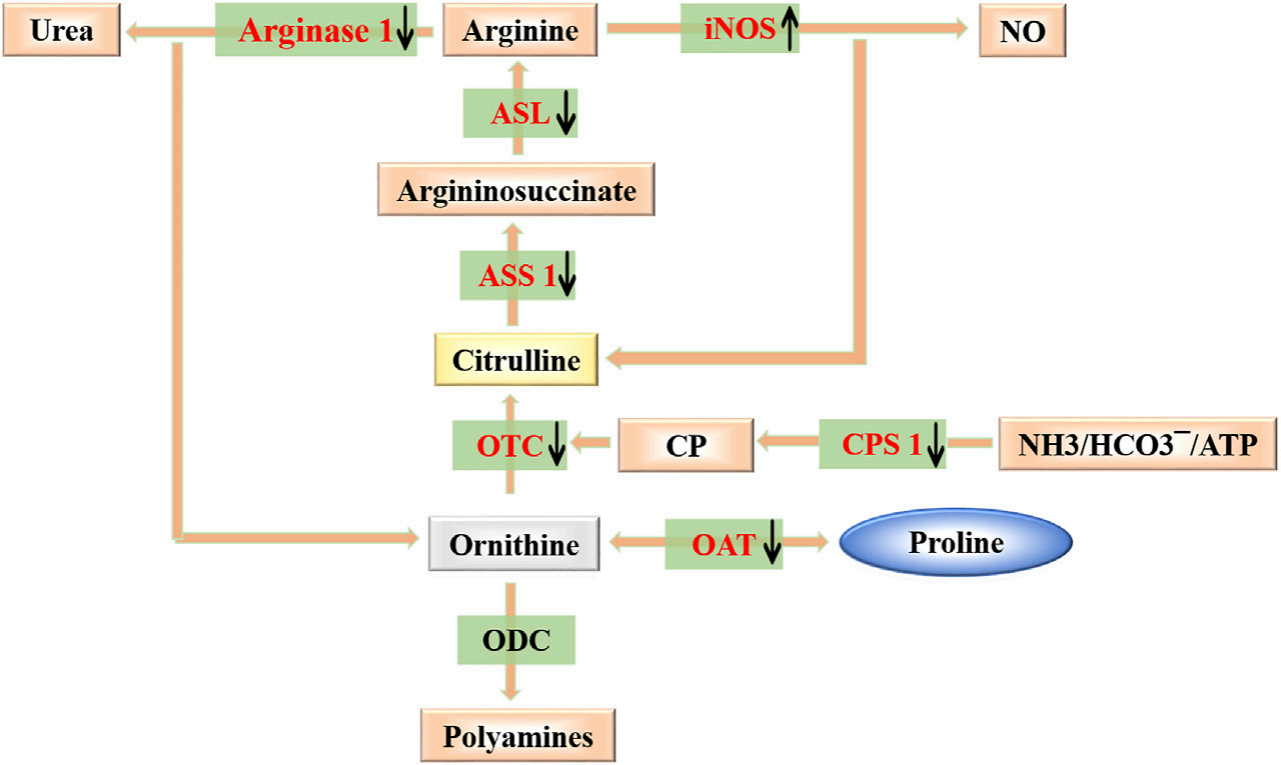
Modulating arginase-ornithine pathway. Scheme for the catabolism of arginine to ornithine/urea or citrulline/NO, production of polyamines and anabolism and catabolism of proline. Also shown is the recycling of citrulline into arginine. Targets of TL against PF are colored in red.

In the present study, we analyzed the efficacy and molecular mechanism of TL in lung fibrosis, using the model of bleomycin-induced fibrosis in mice. PF has characteristic histologic appearances, usually accompanied with extracellular matrix accumulation and remodeling of lung interstitium, which ultimately impair gas exchange (Lederer and Martinez, 2018; Hewitt and Maher, 2019; Upagupta et al., 2018). We observed the body weight, lung index, lung hydroxyproline (HYP) content and lung histology in mice. Compared with the control group, the HYP level and lung index increased in the BLM group (Figure 2). WB and RT-PCR analysis showed the expression of α-smooth muscle actin (α-SMA), *Collagen1* (*Col1a1*) and *Fibronectin* (*Fn1*) were significantly increased in BLM-induced mice (Figure 2). But after TL treatments for 2 weeks, the above results were all down-regulated. Meanwhile, lung histology showed TL treatments significantly decreased lung inflammatory and fibrotic lesions compared with BLM group (Figure 2). Our study demonstrated that TL treatments effectively reduce the deposition of collagen in the pulmonary fibrosis induced by bleomycin in mouse model. We further analyzed the proteomics results and found the beneficial effect of TL are related to the down-regulation of arginase-ornithine pathway (Figures 3–8). However, a kind of senecionine which is hepatotoxic has been found in the TL extract (Supplementary Table S1). Thus we checked relevant reports and confirmed that the minimum toxic dose of senecionine was 10-folds higher than which detected in the TL extract (Schoental et al., 1970). In addition, previous clinical tests on TL confirmed that patients had a good prognosis without poisoning, which reflected the safety of TL to a certain extent (Qiu et al., 2000; Qiu et al., 2001; Mai and Xia, 2009; Ye et al., 2014; Wang and Zhu, 2017). Although the content in the TL extract is much lower than the minimum toxic dose, the use of TL should be carried out rationally with quality control.

Arginase is an enzyme that catalyzes the crucial step in the urea cycle, which can metabolize arginine into urea and ornithine, and dispose toxic ammonia in the body (Wu and Morris, 1998; Morris et al., 2016; Caldwell et al., 2018). It is highly expressed in the liver and is also expressed in some other organs. Both elevated and decreased arginase levels cause many diseases (Jung et al., 2014; Yao et al., 2017). Several studies have confirmed that there was a closed relationship between arginase and fibrogenesis. The content of arginase significantly increased in either patients with PF or in bleomycin-induced mouse fibrosis models (Endo et al., 2003; Kitowska et al., 2008; Ding et al., 2019). Other groups also found increased levels of arginine metabolites creatine, hydroxyproline, and spermidine in human PF lung tissue (Zhao et al., 2017). Upregulated expression of citrulline, ornithine, proline was induced by bleomycin in mice of peripheral blood on Day 28 (Gao et al., 2019). These studies have confirmed increase of arginine metabolism may imply the development of pulmonary fibrosis. In arginase-ornithine pathway, ornithine could be further converted to proline and polyamines through ornithine aminotransferase (OAT) and ornithine decarboxylase (ODC). Proline is a precursor for the synthesis of HYP, which is a main amino acid of collagen synthesis in fibrotic lung tissues (Chow et al., 2018; Li and Wu, 2018). And polyamines play an essential role in cell proliferation and growth, and they also participate in inflammation, wound healing and tissue repair (Halper and Kjaer et al., 2014; Bae et al., 2018; Zhang and Liu, 2018). As we know, collagen deposition and the excessive proliferation of fibroblasts are hallmarks of fibrosis (Mak and Mei, 2017; Michalik et al., 2018; Piersma and Bank, 2019). The supply of them could be a key factor in the process of fibrosis. The abundance of arginase, as well as the expression of other enzymes in arginine metabolic cycle, constitutes essential determinants of proline and polyamines levels, and further affects the progress of fibrosis. Thus, the purpose of present study is to determine if TL could confer protection against development of PF through changing the level of arginase-ornithine pathway in the lung tissue.

We addressed the expression of enzymes in arginase-ornithine pathway *in vivo*. Our data confirmed the results from previous studies of arginase in lung fibrosis (Endo et al., 2003; Kitowska et al., 2008). The expression of arginase in the lungs of fibrotic mice observed in this study is consistent with the collagen deposition induced by BLM. We found that BLM injury was accompanied by a high level of Arg1 expression, which contributed to the accumulation of ornithine. Upregulation of OAT in BLM-induced lung fibrosis mice further produced high level of proline by metabolizing excessive ornithine (Figure 7). Augmenting arginase levels were thought to enhance the fibrosis by accelerating the collagen production. Therefore, treatments with TL resulted in downregulation of Arg1 and OAT expression, which in turn reduced the supply of proline for the synthesis of collagen, thereby inhibiting the progress of fibrosis. Furthermore, the abundance of its substrate, arginine, was also very important. Ornithine can not only be hydrolyzed into proline, but also can be converted into citrulline through ornithine carbamoyltransferase (OTC) and carbamoy-phosphate synthase (CPS1). Argininosuccinate synthase (ASS1) and argininosuccinate lyase (ASL) can further convert citrulline into arginine. OTC, CPS1, ASS1, and ASL were part of arginase-ornithine pathway. The expression of these enzymes affected the content of arginine (Caldwell et al., 2018). We found the expression of OTC, CPS1, ASS1, and ASL was highly upregulated in BLM-induced fibrosis, and these enzymes were also downregulated after treatment with TL (Figure 7). And as we know that bleomycin-induced mouse models are accompanied by early inflammatory injury. Lung injury involves the body in expressing inducible nitric oxide synthase (iNOS) (Caldwell et al., 2018). iNOS is a key mediator of immune activation and inflammation, which produces nitric oxide (NO) and citrulline (Cinelli et al., 2020). Although there will be inflammation after BLM injection, this inflammation will gradually decline as the fibrosis progresses accompanied by excessive wound repair. Proteomics results showed that TL treatment can regulate inflammatory response and immune system (Figure 5), but how it regulates is unclear. In addition to the crucial role of arginine in the arginase-ornithine pathway for formation of proline and polyamines, arginine is also the substrate for iNOS. Therefore, we can regard Arg1 and iNOS as a competitive relationship for arginine (Caldwell et al., 2018). Our work has proved that the expression of Arg1 was increased and the expression of iNOS was inhibited in lung tissues of mice (Figure 7). The increased Arg1 and the decreased iNOS lead to a decrease in NO synthesis, which indicated NOS uncoupling. NO and superoxide (O_2_^.−^) combine to produce oxidant peroxynitrite (ONOO^−^) in coupling (Caldwell et al., 2018). Uncoupling leads to NO and ONOO^−^ reduction. Due to NO and ONOO^−^ play important roles in immunity, the increased Arg1 and the decreased iNOS will lead to changes in immune function (Lee et al., 2003; Popovic et al., 2007). This provided the possibility for fibroblasts to continue to proliferate by evading immunity. In addition, in lung tissues, iNOS can also be regarded as a marker of M1 macrophages, while Arg1 is usually regarded as a marker of M2 macrophages. M1 macrophages initiate early inflammatory responses (Dall’Asta et al., 2012; Das et al., 2015). They undergo dynamic changes during different states of wound repair (Das et al., 2015). Therefore, M2 macrophages with anti-inflammatory effects participate in damage repair and play a more important role in fibrosis (Laskin et al., 2019). With the progression of fibrosis, the ratio of inflammation and wound repair gradually becomes unbalanced, which eventually leads to Arg1 having an advantage in the competition. TL treatments have the ability to balance the relationship between Arg1 and iNOS.

Due to the inhibitory effect of TL on arginase-ornithine pathway, we can regard TL as a new type drug that inhibit arginase. Despite a grow number of drugs have been tested as arginase inhibitors, there are still many limitations. Many arginase inhibitors did not have an ideal application due to their low potency and many side effects (Morris et al., 2009). Although several arginase inhibitors are now available for preclinical use and several have been used for clinical use, many clinical studies have been limited to small-scale “proof-of-concept” studies (Caldwell et al., 2018). In contrast, one of the advantages of TL is that TL has been widely used in clinical practice and has achieved good effects. Now, the search for arginase inhibitors is continuing and involves plant extract testing (Steppan et al., 2013). Plant extracts have great potential to be applied as arginase inhibitors, and several studies have confirmed that most of them are polyphenols (Dal-Ros et al., 2012; Bordage et al., 2017). The composition analysis of TL showed that it has ingredients such as Chlorogenic acid, which are polyphenols. It further confirmed the advantage of TL as a natural herbal formula to inhibit arginase. In addition, as an herbal formula, TL has the advantage of multi-target synergistic treatment. The inhibition of TL on arginase does not only target arginase, but is based on regulating the whole arginase-ornithine cycle. And TL can also regulate iNOS to prevent the balance of Arg1 and iNOS from being disrupted. It avoids the negative effect of only regulating arginase on the cycle. Thus our work suggested that TL can reduce arginase-ornithine pathway to treat pulmonary fibrosis, and may also be used as a new potential drug that inhibit arginase to treat other diseases. Therapies for the metabolism of lung tissue may provide a different perspective for the treatment of pulmonary fibrosis.

## Conclusions

In this study, the underlying action mechanism of TL in BLM-induced pulmonary fibrosis was explored by combining proteomics analysis and experimental validation. In summary, TL can effectively alleviate BLM-induced pulmonary fibrosis by downregulation of the level of arginase-ornithine pathway. It means that the regulation of arginine metabolism may provide a target for the clinical treatment of pulmonary fibrosis.

## Data Availability

The datasets presented in this study can be found in online repositories. The names of the repository/repositories and accession number(s) can be found below: PRIDE, PXD024058.
