# Supplementary material for: Tianlongkechuanling Inhibits Pulmonary Fibrosis Through Down-Regulation of Arginase-Ornithine Pathway
Source: Front Pharmacol. 2021 Apr 22;12:661129. doi: 10.3389/fphar.2021.661129 (PMC8114272; doi:10.3389/fphar.2021.661129)
Supplement: Supplementary file 2 [file table1.docx]

**Main chemical components of extract of TL**

| **Peak number** | ***t*R/min** | **Molecular**  **formula** | **Accurate**  **mass[M – H]^-^** | **Calculated**  **mass[M – H]^-^** | **Fragmentation（MS/MS）** | **Error (ppm)** | **Identified**  **compounds** |
| --- | --- | --- | --- | --- | --- | --- | --- |
| 1 | 1.22 | C4H6O5 | 133.0137 | 133.0131 | 115.0030;71.0128 | -4.51 | D-(+)-Malic acid |
| 2 | 3.00 | C7H6O4 | 153.0188 | 153.019 | 109.0287 | 1.31 | 3,4-Dihydroxybenzoic acid |
| 3 | 10.19 | C9H8O3 | 163.0395 | 163.0398 | 119.0496 | 1.84 | p-Coumaric acid |
| 4 | 1.77 | C7H6O5 | 169.0137 | 169.014 | 128.0348 | 1.78 | Gallic acid |
| 5 | 1.22 | C6H6O6 | 173.0086 | 173.0089 | 131.0820;111.0080;93.0337 | 1.73 | trans-Aconitic acid |
| 6 | 8.87 | C11H9NO4 | 218.0454 | 218.0464 | 174.0560;159.0324 | 4.59 | 6-Methoxykynurenic acid |
| 7 | 12.12 | C11H12O5 | 223.0607 | 223.062 | 208.0383;164.0477 | 5.83 | Sinapinic acid |
| 8 | 26.39 | C16H12O5 | 283.0607 | 283.0616 | 268.0348;239.0350;184.0537;165.9900;163.0029;135.0062 | 3.18 | Oroxylin A |
| 9 | 21.56 | C16H12O6 | 299.0556 | 299.0581 | 284.0382;269.0444;239.0350;136.9887 | 8.36 | Hispidulin |
| 10 | 17.84 | C16H12O7 | 315.0505 | 315.0526 | 300.0292;178.9574;112.9864 | 6.67 | Pollenitin |
| 11 | 1.17 | C12H22O11 | 341.1084 | 341.1096 | 221.0670;113.0237;89.0234 | 3.52 | Sucrose |
| 12 | 3.33 | C16H18O9 | 353.0873 | 353.0884 | 191.0563;179.0350;135.0446 | 3.12 | 3-O-Caffeoylquinic acid |
| 13 | 12.53 | C27H30O16 | 609.1456 | 609.1485 | 301.03702;300.0292 | 4.76 | Rutin |

| **Peak number** | ***t*R/min** | **Molecular**  **formula** | **Accurate**  **mass[M + H]^+^** | **Calculated**  **mass[M + H]^+^** | **Fragmentation（MS/MS）** | **Error (ppm)** | **Identified**  **compounds** |
| --- | --- | --- | --- | --- | --- | --- | --- |
|  | 6.43 | C8H11N | 122.0969 | 122.0971 | 111.8166;105.0707;96.0452;80.0504. | 1.64 | Phenethylamine |
|  | 1.54 | C6H6N2O | 123.0558 | 123.056 | 96.0452;80.0504 | 1.63 | Nicotinamide |
|  | 11.50 | C10H14 | 135.1174 | 135.1174 | 107.0863;93.0708;62.9827 | 0.00 | p-Cymene |
|  | 1.53 | C5H4N4O | 137.0463 | 137.0468 | 101.5133;93.2170;70.2568 | 3.65 | Hypoxanthine |
|  | 17.18 | C10H12O | 149.0966 | 149.0968 | 121.1090;93.0708 | 1.34 | Estragol |
|  | 6.44 | C9H13NO | 152.1075 | 152.1073 | 134.0971;117.0707 | -1.31 | D-Cathine |
|  | 7.58 | C10H15NO | 166.1232 | 166.1235 | 148.1127 | 1.81 | (-)-Ephedrine |
|  | 12.09 | C11H14O2 | 179.1072 | 179.1073 | 162.1285;133.1019;107.0863 | 0.56 | Methyleugenol |
|  | 1.85 | C10H13NO2 | 180.1023 | 180.1027 | 163.0760;145.0654;85.0293 | 2.22 | Salsolinol |
|  | 10.77 | C10H13NO2 | 180.1023 | 180.1027 | 162.0921;138.0920;112.0765;69.0345 | 2.22 | (-)-Salsolinol |
|  | 8.74 | C11H17NO | 180.1388 | 180.1391 | 162.1286;135.0811;117.0707 | 1.67 | Methylephedrine |
|  | 1.81 | C10H17NO3 | 200.1286 | 200.1291 | 154.1597;128.9514;86.0974 | 2.50 | Tussilagine |
|  | 29.01 | C12H16O3 | 209.1177 | 209.117 | 191.1073;163.0761;135.0811;117.0707 | -3.35 | beta-Asarone |
|  | 19.63 | C13H22O2 | 211.1698 | 211.1694 | 193.1595;175.1490;137.0967;109.1020;69.0709 | -1.89 | alpha-Cyclogeraniol acetate |
|  | 14.18 | C13H14O3 | 219.1021 | 219.1025 | 189.0918;159.0811;131.0862;82.9458 | 1.83 | Hydroxytremetone |
|  | 6.63 | C12H17NO3 | 224.1286 | 224.1291 | 206.1186;147.1050;90.0559 | 2.23 | Maokonine |
|  | 15.53 | C13H20O3 | 225.149 | 225.149 | 165.1282;111.0813;97.0656 | 0 | (+)-Blumenol A |
|  | 42.05 | C15H20O2 | 233.1541 | 233.1546 | 215.1437;187.1488;145.1018;107.0863 | 2.14 | Cauleslactone |
|  | 25.64 | C15H10O4 | 255.0657 | 255.0661 | 177.0918;163.0760;135.0810;107.0863 | 1.57 | Chrysin |
|  | 17.57 | C15H12O4 | 257.0814 | 257.0811 | 239.0713;137.0239;113.0605 | -1.17 | Pinocembrin |
|  | 17.46 | C15H12O5 | 273.0763 | 273.0768 | 169.0139;131.0498;123.0813 | 1.83 | Dihydrobaicalein |
|  | 26.39 | C16H12O5 | 285.0763 | 285.0769 | 270.0534 | 2.10 | Wogonin |
|  | 14.20 | C15H10O6 | 287.0555 | 287.0564 | 153.019 | 3.14 | Scutellarein |
|  | 12.32 | C15H12O6 | 289.0712 | 289.072 | 169.0140;147.0448;90.9776 | 2.77 | Eriodictyol |
|  | 21.55 | C16H12O6 | 301.0712 | 301.0721 | 286.0485 | 2.99 | Scutevulin |
|  | 26.73 | C16H12O6 | 301.0712 | 301.072 | 209.1180;181.1231;163.0761;135.0811;107.0863 | 2.66 | Takakin |
|  | 10.90 | C15H12O7 | 305.0661 | 305.0668 | 287.0565;259.0613;241.0506;215.0712 | 2.29 | 3,5,7,2',5'-Pentahydroxyflavanone |
|  | 26.47 | C17H14O6 | 315.0868 | 315.0875 | 300.0640;169.0865;74.0974 | 2.22 | Skullcapflavone I |
|  | 10.42 | C20H27NO3 | 330.2069 | 330.2078 | 171.1135;70.0661 | 2.73 | Trilostane |
|  | 16.21 | C17H14O7 | 331.0818 | 331.0827 | 316.0592 | 2.72 | Malvidin |
|  | 16.04 | C19H27NO6 | 366.1916 | 366.1925 | 253.1534;168.1027 | 2.46 | Senkirkine |
|  | 26.29 | C19H18O8 | 375.108 | 375.1091 | 345.0619;327.0513;227.0556 | 2.93 | Skullcapflavone II |
|  | 14.73 | C21H20O9 | 417.1185 | 417.1182 | 318.0984;351.0877;297.0769;267.0662 | -0.72 | Baicalein 7-rhamnoside |
|  | 13.15 | C21H22O10 | 435.1291 | 435.1308 | 387.0837;273.0769;153.0190 | 3.91 | 5,7,2'-Trihydroxyflavanone 7-glucoside |
|  | 19.28 | C21H18O11 | 447.0927 | 447.0937 | 285.0769;271.0613 | 2.24 | Baicalin |
|  | 18.75 | C22H22O10 | 447.1291 | 447.1306 | 285.0769;271.0613 | 3.35 | Baicalein 6-methyl ether 7-glucoside |
|  | 17.39 | C22H20O12 | 477.1033 | 477.105 | 301.072 | 3.56 | Hispidulin 7-O-beta-glucuronide |
|  | 16.20 | C23H24O12 | 493.1346 | 493.1363 | 331.0827 | 3.45 | 5,2',6'-Trihydroxy-6,7-dimethoxyflavone 2'-glucoside |
|  | 15.13 | C24H26O13 | 523.1451 | 523.1473 | 361.0934 | 4.21 | 5,2'-Dihydroxy-7,8,6'-trimethoxyflavanone 2'-O-glucuronide |
|  | 10.99 | C26H28O14 | 565.1557 | 565.1579 | 379.0828;325.0719;295.0613 | 3.89 | 6-C-Xylopyranosyl-8-C-galactopyranosylapigenin |
|  | 12.52 | C27H30O16 | 611.1612 | 611.1638 | 303.0511;85.0293 | 4.25 | Kaempferol 3,7-O-beta-D-diglucopyranoside |
|  | 18.16 | C27H30O16 | 611.1612 | 611.1636 | 317.0671;147.0448 | 3.93 | Herbacetin 3-rhamnoside-8-glucoside |
|  | 14.26 | C21H20O11 | 449.1084 | 449.1097 | 303.0512;287.0563;85.0293;71.0502 | 2.89 | Cynaroside |
|  | 7.77 | C16H18O9 | 355.1029 | 355.1036 | 166.1026;163.0396 | 1.97 | Chlorogenic acid |
